# The neutrophil percentage-to-albumin ratio is associated with all-cause mortality in patients with chronic heart failure

**DOI:** 10.1186/s12872-023-03472-9

**Published:** 2023-11-18

**Authors:** Xin Wang, Yuan Zhang, Yuxing Wang, Jia Liu, Xiaorong Xu, Jiamei Liu, Mulei Chen, Linying Shi

**Affiliations:** Heart Center and Beijing key laboratory of hypertension research, Beijing Chaoyang Hospital, Capital Medical University, 8# Gong-Ti South Road, 100020 Beijing, China

**Keywords:** Neutrophil percentage-to-albumin ratio (NPAR), Chronic heart failure, All-cause mortality

## Abstract

**Background:**

In this study, we evaluated the predictive utility of neutrophil percentage-to-albumin ratio (NPAR) for all-cause mortality in patients with chronic heart failure (CHF).

**Methods:**

Patients diagnosed as CHF enrolled in this retrospective cohort study were from Beijing Chaoyang Hospital, capital medical university. Admission NPAR was calculated as neutrophil percentage divided by serum albumin. The endpoints of this study were defined as 90-day, 1-year and 2-year all-cause mortality. Multivariable Cox proportional hazard regression model was performed to confirm the association between NPAR and all-cause mortality. Receiver operating characteristics (ROC) curves were used to evaluate the ability for NPAR to predict all-cause mortality.

**Results:**

The 90-day (*P* = 0.009), 1-year (*P* < 0.001) and 2-year (*P* < 0.001) all-cause mortality in 622 patients with CHF were increased as admission NPAR increased. Multivariable Cox regression analysis found the higher NPAR value was still independently associated with increased risk of 90-day (Group III versus Group I: HR, 95% CI: 2.21, 1.01–4.86, *P* trend = 0.038), 1-year (Group III versus Group I: HR, 95% CI:2.13, 1.30–3.49, *P* trend = 0.003), and 2-year all-cause mortality (Group III versus Group I: HR, 95% CI:2.06, 1.37–3.09, *P* trend = 0.001), after adjustments for several confounders. ROC curves revealed that NPAR had a better ability to predict all-cause mortality in patients with CHF, than either albumin or the neutrophil percentage alone.

**Conclusions:**

NPAR was independently correlated with 90-day, 1-year, and 2-year all-cause mortality in patients with CHF.

## Background

Heart failure (HF) is a complex clinical syndrome with symptoms and signs that result from any structural or functional impairment of ventricular filling or ejection of blood. A recent U.S. evaluation found total deaths caused by HF have increased from 275,000 to 2009 to 310,000 in 2014 [1]. Most of the HF patients have a prolonged and deteriorative course, which is defined as chronic heart failure (CHF). CHF is growing health and economic burden in the entire world. A report examining the Chinese population found age-adjusted incidence of CHF was 1.10%, with 275 per 100,000 individuals per year. Hospitalization cost and outpatient cost per capita of patients with HF were $4,406.8 and $892.3. And the proportion of hospitalization ≥ 3 times was 40.5% [2]. CHF has the poor prognosis,and identifying sensitive prognostic indicators of CHF can help medical for discriminating high-risk patients to help implement appropriate treatment. Therefore, the impact of early biomarkers on the prognosis of CHF is worth further investigation.

The pathogenesis of CHF has not been fully elucidated, which involves complex pathophysiological processes. The mechanism may be related to systemic inflammation [3, 4]. Neutrophil, producing inflammatory mediators such as chemokines and cytokines, plays an important role in mediating inflammatory responses [5]. Albumin, as a crucial regulatory protein, is involved in anti-inflammatory, antioxidant, anticoagulant and antiplatelet aggregation activity as well as colloid osmotic effect [6–10]. It is now well established that hypoalbuminemia is a potent prognosticator independent of other risk factors in patients with CHF [11].

NPAR, calculated as neutrophil percentage numerator divided by serum albumin concentration, can amplify the changes of these two accessible evaluation parameters.

Recently, several studies showed that the NPAR, as an inflammation-based prognostic predictor, was associated with clinical outcomes in patients with ST-segment elevation myocardial infarction(STEMI) [12], cardiogenic shock(CS) [13], acute kidney injury (AKI) [14] and septic shock [15]. However, to our knowledge, there was no study exploring the influence of NPAR on the outcomes of patients with CHF. The purpose of this study is to investigate the association between the admission NPAR level and all-cause mortality in patients with CHF.

## Materials and methods

### Study design and population

We retrospectively enrolled 622 patients with CHF who were admitted to the department of cardiology, Beijing Chao-yang Hospital, Capital Medical University from January 2011 to December 2016. The definition of CHF is the presence of heart failure symptoms and/or signs, with the increase of N-terminal pro brain natriuretic peptide (NT-proBNP), with/without the reduction of left ventricular ejection fraction (LVEF), according to the European Society of Cardiology (ESC) guidelines for the diagnosis and treatment of acute and chronic heart failure [16]. According to the guidelines, CHF can be divided into three types: heart failure with preserved (HFpEF), mid-range (HFmrEF) and reduced ejection fraction (HFrEF).The severity of enrolled CHF patients must meet the criteria of medium-risk patients with worsening heart failure: (1) New York Heart Association (NYHA) grade III-IV; (2)the baseline NT-proBNP > 1000 pg/mL;(3) 6-minute walk test < 150 m [17, 18]. Patients were excluded because of the complications of acute or chronic infectious diseases, tumor, autoimmune diseases, hepatobiliary disorders, hematological proliferative diseases or had no complete records. This study was approved by the Ethical Committee of Beijing Chaoyang Hospital, Capital Medical University and was conducted in accordance with the Declaration of Helsinki. Free and explicit terms of consent were obtained from all participants.

### Clinical and heart function assessment

Demographic information and cardiovascular risk factors, including age, gender, coronary artery disease (CAD), hypertension, diabetes, hyperlipidemia, atrial fibrillation (AF), smoking history and operation history, were retrospectively collected. Systemic blood pressure (SBP), diastolic blood pressure (DBP) and heart rate (HR) were recorded on admission first. All the patients underwent routine echocardiography within 48 h after admission, using the VV5 ultrasound device. LVEF was calculated by Simpson method to quantitatively evaluate the left ventricular systolic function. During hospitalization period, all the patients received standard pharmacological therapy (diuretic, digoxin, angiotensin-converting enzyme inhibitors (ACEI)/angiotensin receptor blockers (ARB), β-blockers and spironolactone, unless these agents were contraindicated), according to the established guidelines.

### Laboratory analysis and NPAR calculation

Blood samples were collected from the antecubital vein, on the first morning after admission. Routine complete blood count, blood biochemistry parameters, cardiac markers including NT-proBNP and cardiac troponin I (cTnI) were measured by an automatic analyzer according to the hospital protocol at the central chemistry laboratory of Beijing Chao-yang Hospital. Neutrophil percentage was expressed as the percentage of neutrophil in leukocytes, and calculated automatically by the analyzer. Serum albumin level was measured using the bromocresogreen assay with album kits and AU5800 biochemistry analysis system (Beckman Coulter Company), according to the manufacturer’s instructions. The NPAR was calculated as the neutrophil percentage as the numerator divided by albumin using the same blood samples according to the formula: Neutrophil percentage(%)*100%/Albumin(g/dL). Serum glucose (Glu), creatinine (Cr), triglycerides (TG), total and low-density lipoprotein-cholesterol (TC and LDL-c) and hypersensitive C-reaction protein (hs-CRP) were evaluated, which went through an overnight fast and quit smoking and drinking.

### Follow-up and endpoint events

All subjects were followed up from their first hospitalization to death, or Jan 2019. Endpoint status and causes were determined through outpatient visits, medical records, telephone contacts, and text messages. For deceased patients, death certificates were procured, and the next of kin were interviewed to determine the time of death. The main endpoints in the study were 90-day, 1-year, and 2-year all-cause mortality. All endpoint events were adjudicated by members of the independent Endpoint Committee, who were unaware of the study group.

### Statistical analysis

Continuous variables are presented as mean and standard deviation or as median and quartiles where appropriate. Categorical variables were expressed as frequencies with percentages. Continuous variables were compared with the Kruskal-Wallis test. Pearson’s χ2 test or Fisher’s exact test was used for categorical variables as appropriate. The Kaplan-Meier (KM) curves were used to plot unadjusted survival rates and the log-rank test was used to compare differences between the three NPAR groups.

Cox proportional hazards regression models were used to calculate hazard ratios (HR) with 95% confidence intervals (CI) in order to evaluate the independent effect of NPAR for 90-day, 1-year, and 2-year all-cause mortality. Model I was adjusted for the confounders age, gender, Model II was adjusted for the confounders age, gender, CAD, hypertension, diabetes, hyperlipidemia, chronic kidney disease (CKD), AF, percutaneous transluminal coronary intervention (PCI), coronary artery bypass grafting (CABG).

Receiver operating characteristics (ROC) curves were constructed to evaluate the sensitivity and specificity of admission NPAR, neutrophil and albumin. And the area under the curve (AUC) was used to estimate the accuracy of admission NPAR, neutrophil and albumin, which was as a predictor for 90-day, 1-year, and 2-year all-cause mortality. All tests were two-tailed and *P* < 0.05 was considered to indicate a statistical significance. All statistical analyses were performed using IBM SPSS Statistical Software for Windows 26.0 (IBM Corp.).

## Results

### Subject characteristics

A total of 622 patients with CHF were enrolled in our study. According to the tertiles of admission NPAR, they were divided into three groups (Group I: NPAR ≤ 18.0; Group II: 18.0<NPAR<21.2; Group III: NPAR ≥ 21.2). The baseline characteristics are presented in Table 1. Patients in the highest tertile of NPAR level had lower BMI, hemoglobin, albumin, and higher values of BP, HR, Cr, uric acid (UA) and hs-CRP. Moreover, they had more comorbidities of CAD and CKD than the other two groups. In addition, left ventricular function was more compromised in patients with the highest tertile of NPAR as indicated by a higher NT-proBNP level along with lower usage of β-blockers and ACEIs/ARBs (*P* < 0.05).

**Table 1:** Baseline characteristics of patients with CHF among three NPAR groups

Characteristics	Total	Group I (NPAR ≤ 18.0)	Group II (18.0 < NPAR < 21.2)	Group III (NPAR ≥ 21.2)	P value
Number	622	209	207	206	
NPAR	19.7(17.0,22.6)	16.0(14.3,17.0)	19.7(18.8,20.4)	24.7(22.6,27.5)	< 0.001
**Demographics**					
Age, years	70.0(58.0,77.0)	68.0(57.0,76.0)	69.0(58.0,77.0)	71.5(60.0,78.0)	0.100
Gender, male, n(%)		122(58.4)	132(63.8)	129(62.6)	0.491
BMI, kg/m^2^	24.8(22.0,27.5)	25.1(22.2,27.8)	25.4(22.4,27.9)	23.8(21.2.26.5)	0.002
**History of disease**					
Hypertension	435(69.9)	145(69.4)	139(67.1)	151(73.3)	0.386
Diabetes	280(45.0)	89(42.6)	92(44.4)	99(48.1)	0.523
Hypercholesterolemia	338(54.3)	122(58.4)	106(51.2)	110(53.4)	0.323
CAD	394(63.3)	129(61.7)	120(58.0)	145(70.4)	0.027
AF	236(37.9)	83(39.7)	79(38.2)	74(35.9)	0.726
Smoking	315(50.6)	108(51.7)	103(49.8)	104(50.5)	0.925
CKD	169(27.2)	33(15.8)	46(22.2)	90(43.7)	< 0.001
PCI	140(22.5)	43(20.6)	45(21.7)	52(25.2)	0.496
CABG	80(12.9)	26(12.4)	27(13.0)	27(13.1)	0.115
**Physical examination**					
SBP, mmHg	130.0(115.0,150.0)	130.0(112.5,140.0)	130.0(115.0,150.0)	135.0(120.0,154.0)	0.043
DBP, mmHg	80.0(70.0,90.0)	76.0(67.0,83.5)	80.0(70.0,90.0)	80.0(70.0,90.0)	0.015
h, bpm	80.0(70.0,97.0)	80.0(65.5,91.5)	80.0(70.0,98.0)	83.5(70.0,100.0)	0.018
**Laboratory tests**					
Neutrophil percentage,%	67.3(61.0,73.2)	57.7(52.2,62.3)	68.1(64.7,71,6)	74.7(70.0,80.8)	< 0.001
Albumin, g/L	33.9 ± 4.7	37.0 ± 3.8	34.8 ± 2.8	30.0 ± 4.1	< 0.001
Hemoglobin, g/dl	125.0(107.0,137.0)	130.0(112.0,143.0)	128.0(114.0,138.0)	116.0(100.0,131.2)	< 0.001
Creatinine, umol/L	97.9(78.8,134.9)	89.0(74.9,107.4)	94.6(76.8,125.2)	122.7(88.9,205.0)	< 0.001
Uric acid, umol/L	410.6(333.6,504.1)	388.3(326.4,472.8)	412.2(336.8,507.0)	430.7(347.5,540.8)	0.021
HDL-c, mmol/L	1.04(0.85,1.24)	1.03(0.84,1.20)	1.02(0.85,1.24)	1.07(0.86,1.29)	0.434
LDL-c, mmol/L	2.09(1.60,2.65)	2.16(1.60,2.77)	2.00(1.59,2.50)	2.10(1.58,2.63)	0.277
Troponin I, ng/ml	0.03(0.0.09)	0.03(0,0.06)	0.03(0,0.09)	0.03(0,0.11)	0.423
NT-proBNP, pg/ml	3277.0(1540.0,7950.0)	2065.0(994.2,3991.0)	3198.0(1596.0,6971.8)	7162.0(2926.8,15760.8)	< 0001
HbA_1_c,%	6.4(5.9,7.2)	6.4(5.9,7.2)	6.5(5.9,7.3)	6.4(6.0,7.1)	0.738
Hs-CRP	6.7(2.5,12.4)	3.4(1.2,9.6)	7.4(3.2,12.6)	10.5(4.0,13.4)	< 0.001
**Ultrasound**					
LVEDD, mm	56.0(50.0,63.0)	57.0(50.0,63.5)	56.0(50.0,65.0)	55.5(50.8,62.0)	0.705
LVESD, mm	44.0(34.0,52.0)	45.0(32.3.52.0)	44.0(33.0,53.0)	43.0(35.0,50.3)	0.969
LVEF,%	43.0(33.0,63.0)	44.0(35.0,61.5)	44.0(34.0,60.0)	41.5(32.0,58.0)	0.116
**Medication**					
β-blockers	374(60.1)	141(67.5)	124(59.9)	109(52.9)	0.010
ACEIs/ARBs	343(55.1)	123(58.9)	124(59.9)	96(46.6)	0.010
MRA	460(74.0)	161(77.0)	154(74.4)	145(70.4)	0.300
Statins	369(59.3)	125(59.8)	118(57.0)	126(61.2)	0.680
Digoxin	354(56.9)	110(52.6)	124(59.9)	120(58.3)	0.291

Abbreviation: NPAR: neutrophil percentage-albumin ratio; BMI: body mass index; CAD: coronary artery disease; AF: atrial fibrillation; CKD: chronic kidney disease; PCI: percutaneous coronary intervention; CABG: coronary artery bypass grafting; SBP: systolic blood pressure; DBP: diastolic blood pressure; HR: heart beat; HDL-C: high-density lipoprotein cholesterol; LDL-C: low-density lipoprotein cholesterol; NT-proBNP: N-terminal pro brain natriuretic peptide; Hs-CRP: high-sensitivity C-reactive protein; LVEDD: left ventricular end-diastolic dimension; LVESD: left ventricular end-systolic dimension; LVEF: left ventricular ejection fraction; ACEIs: angiotensin-converting enzyme inhibitors; ARBs: angiotensin receptor blockers; MRA: mineralcorticoid receptor antagonist

### Admission NPAR and outcomes

**Fig. 1:**
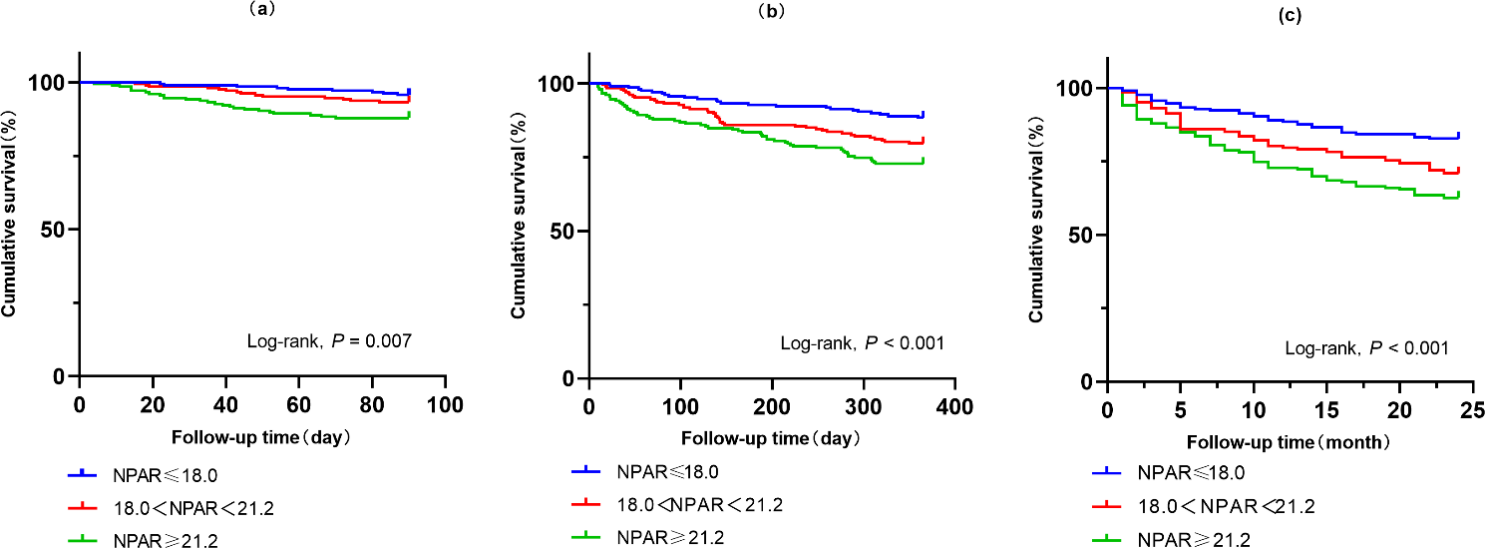
(**a**) Kaplan-Meier curves showing the association between the NPAR and 90-day all-cause mortality. (**b**) Kaplan-Meier curves showing the association between the NPAR and 1-year all-cause mortality. (**c**) Kaplan-Meier curves showing the association between the NPAR and 2-year all-cause mortality. NPAR: neutrophil percentage-albumin ratio

The clinical outcomes of the subjects across the tertiles of NPAR are shown in Table 2. The overall 90-day, 1-year and 2-year all-cause mortality were 7.7%, 19.6%, and 27.8%, respectively. Moreover, as the admission NPAR levels increased, the all-cause death rates of 90-day, 1-year and 2-year were all distinctly increased. Kaplan-Meier curves of 90-day (Log rank, *P* = 0.007), 1-year (Log rank, *P* < 0.001), and 2-year (Log rank, *P* < 0.001) all-cause mortality stratified by the tertiles of admission NPAR are shown in Fig. 1. Similarly, the 90-day, 1-year and 2-year all-cause mortality in the highest NPAR level group (Group III) were significantly higher than the other two groups, which showed that a higher NPAR value was significantly associated with a worse outcome.

**Table 2:** The all-cause mortality among the three NPAR groups

Outcomes	Total	Group I (NPAR ≤ 18.0)	Group II (18.0 < NPAR < 21.2)	Group III (NPAR ≥ 21.2)	*P* value
Number	622	209	207	206	
90-day mortality, n(%)	48(7.7)	9(4.3)	14(6.8)	25(12.1)	0.009
1-year mortality, n(%)	122(19.6)	24(11.5)	42(20.3)	56(27.2)	< 0.001
2-year mortality, n(%)	173(27.8)	36(17.2)	60(29.0)	77(37.4)	< 0.001

Abbreviation: NPAR: neutrophil percentage-albumin ratio

### Prognostic value of admission NPAR for all-cause mortality

The independent effect of admission NAPR on all-cause mortality among patients with CHF was explored by Cox regression models. The results are summered in Table 3. Group I (NPAR ≤ 18.0) was considered as the reference group. In the univariable Cox regression analysis, higher admission NPAR was associated with increased risk of all-cause mortality. Furthermore, in model I, after adjustments for age and gender, patients with the highest NPAR had the highest risk of 90-day, 1-year and 2-year all-cause mortality (Group III versus Group I: HR, 95% CI: 2.73, 1.27–5.86, *P* trend = 0.010; 2.45, 1.52–3.96, *P* trend < 0.001; 2.29, 1.54–3.41, *P* trend < 0.001), compared with the reference group. When examined as continuous variables in model I, each unit’s higher NPAR was associated with increased 90-day (HR, 95% CI: 1.09, 1.04–1.15; *P* < 0.001), 1-year (HR, 95% CI: 1.08, 1.05–1.11; *P* < 0.001) and 2-year (HR, 95%CI: 1.07, 1.04–1.09; *P* < 0.001) all-cause mortality. In model II, age, gender, CAD, hypertension, diabetes and hyperlipidemia, the history of CRF, AF, PCI and CABG were incorporated into the regression model. A higher NPAR value was still identified as an independent predictor of 90-day, 1-year and 2-year all-cause mortality (Group III versus Group I: HR, 95% CI: 2.21, 1.01–4.86, *P* trend = 0.038; 2.13, 1.30–3.49, *P* trend = 0.003; 2.06, 1.37–3.09, *P* trend = 0.001) in patients with CHF. When examined as continuous variables in model II, each unit’s higher NPAR was still associated with increased 90-day (HR, 95% CI: 1.08, 1.03–1.14; *P* = 0.004), 1-year (HR, 95% CI: 1.07, 1.03–1.10; *P* < 0.001) and 2-year (HR, 95% CI: 1.06, 1.03–1.09; *P* < 0.001) all-cause mortality, independently (Table 3).

**Table 3:** Association among the three NPAR groups and all-cause mortality in patients with CHF

Clinical outcomes	Non-Adjusted			Model I			Model II		
	HR (95% CI)	*P* value	*P* Trend	HR (95% CI)	*P* value	*P* Trend	HR(95%CI)	*P* value	*P* Trend
**90-day mortality**									
NPAR	1.09(1.04, 1.14)	< 0.001		1.09(1.04, 1.15)	< 0.001		1.08(1.03,1.14)	0.004	
Group I (NPAR ≤ 18.0)	1.0(ref)		0.010	1.0(ref)		0.021	1.0(ref)		0.044
Group II (18.0 < NPAR < 21.2)	1.60(0.69, 3.69)	0.273		1.52(0.66, 3.51)	0.330		1.40(0.83,2.27)	0.235	
Group III (NPAR ≥ 21.2)	3.00(1.40, 6.43)	0.005		2.73(1.27, 5.86)	0.010		2.21(1.01,4.86)	0.038	
**1-year mortality**									
NPAR	1.08(1.05, 1.11)	< 0.001		1.08(1.05, 1.11)	< 0.001		1.07(1.03,1.10)	< 0.001	
Group I (NPAR ≤ 18.0)	1.0(ref)		< 0.001	1.0(ref)		0.001	1.0(ref)		0.010
Group II (18.0 < NPAR < 21.2)	1.86(1.13, 3.07)	0.016		1.81(1.10, 2.99)	0.021		1.76(1.06,2.93)	0.029	
Group III (NPAR ≥ 21.2)	2.64(1.64, 4.26)	< 0.001		2.45(1.52, 3.96)	< 0.001		2.13(1.30,3.49)	0.003	
**2-year mortality**									
NPAR	1.07(1.04, 1.09)	< 0.001		1.07(1.04, 1.09)	< 0.001		1.06(1.03,1.09)	< 0.001	
Group I (NPAR ≤ 18.0)	1.0(ref)		< 0.001	1.0(ref)		< 0.001	1.0(ref)		0.002
Group II (18.0 < NPAR < 21.2)	1.79(1.19, 2.71)	0.006		1.74(1.15, 2.63)	0.009		1.70(1.12,2.59)	0.013	
Group III (NPAR ≥ 21.2)	2.49(1.67,3.69)	< 0.001		2.29(1.54,3.41)	< 0.001		2.06(1.37,3.09)	0.001	

Cox proportional hazards regression models were used to calculate hazard ratios (HR) with 95% confidence intervals (CI). Model I was adjusted for the confounders age, gender, Model II was adjusted for the confounders age, gender, CAD, hypertension, diabetes, hyperlipidemia, CRF, AF, PCI and CABG. Abbreviation: NPAR: neutrophil percentage-albumin ratio

We also used Cox proportional hazards regression models to explore the association between NPAR and all-cause mortality in three different classifications of CHF. We found a higher NPAR value was significantly associated with increased 90-day (HR, 95% CI: 1.15, 1.05–1.25; *P* = 0.003), 1-year (HR, 95% CI: 1.08, 1.04–1.14; *P* = 0.004), and 2-year (HR, 95%CI: 1.05, 1.01–1.10; *P* = 0.016) all-cause mortality in HFpEF patients. It was also significantly associated with 2-year all-cause mortality in HFmrEF patients (HR, 95%CI: 1.10, 1.02–1.18; *P* = 0.016), shown in Table 4.

**Table 4:** Association among NPAR and all-cause mortality in three classifications of CHF

	HFpEF		HFmrEF HFrEF			
	HR (95% CI)	*P* value	HR (95% CI)	*P* value	HR(95%CI)	*P* value
**90-day mortality**						
NPAR	1.15(1.05, 1.25)	0.003	1.07(0.93, 1.23)	0.362	1.06(0.97,1.15)	0.240
**1-year mortality**						
NPAR	1.08(1.04, 1.14)	0.004	1.08(0.99, 1.18)	0.085	1.06(0.99,1.12)	0.062
**2-year mortality**						
NPAR	1.05(1.01, 1.10)	0.016	1.10(1.02, 1.18)	0.016	1.04(0.99,1.10)	0.094

Abbreviation: NPAR: neutrophil percentage-albumin ratio; HFpEF: heart failure with preserved ejection fraction; HFmrEF: heart failure with mid-range ejection fraction; HFrEF: heart failure with reduced ejection fraction; CHF: chronic heart failure

**Fig. 2:**
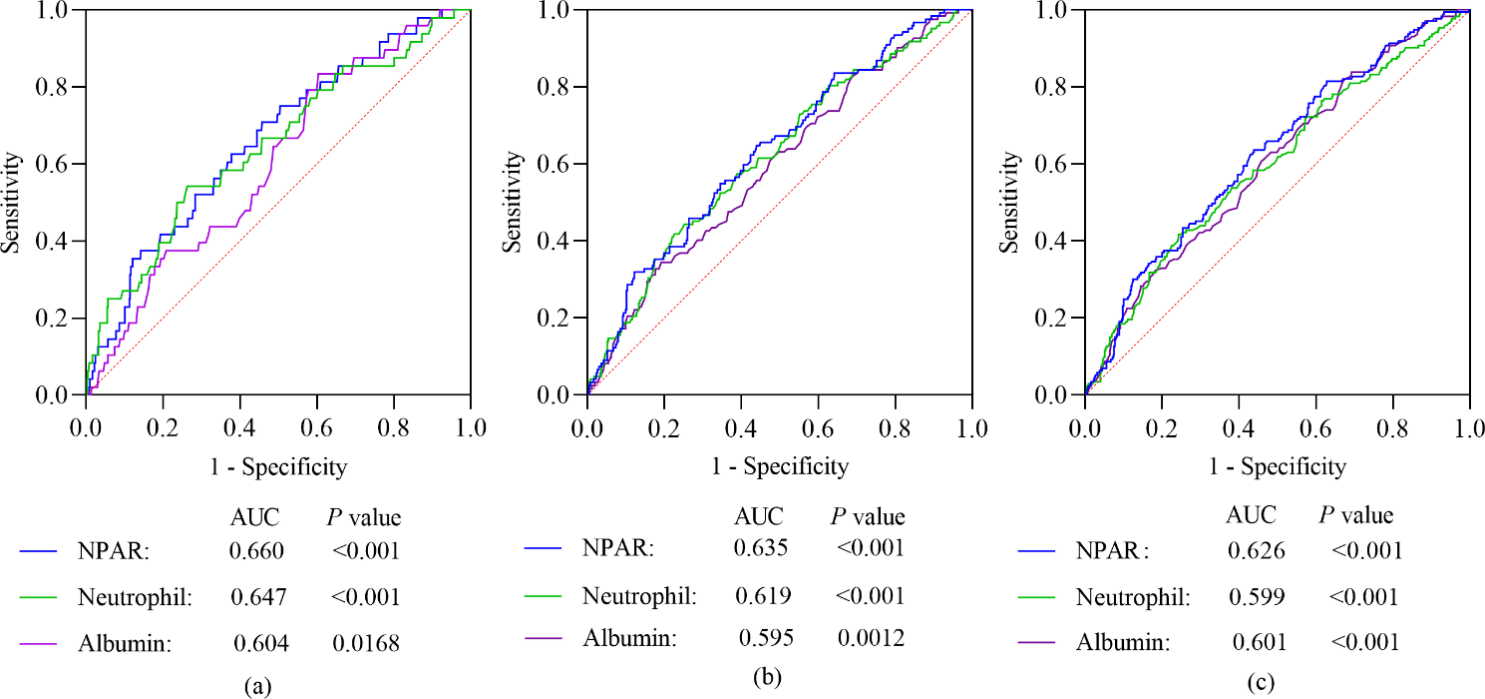
ROC curves of NPAR, neutrophil percentage and albumin for prediction of all-cause mortality. (**a**) About 90-day all-cause mortality. (**b**) About 1-year all-cause mortality. (**c**) About 2-year all-cause mortality. NPAR value was a more effective marker for predicting all-cause mortality in patients with CHF. NPAR: neutrophil percentage-albumin ratio; AUC: area under the curve

ROC curves were used to verify the ability of NPAR for predicting all-cause mortality, compared with neutrophil percentage and albumin, separately. The results are shown in Fig. 2. The AUCs of NPAR, neutrophil percentage and albumin for 90-day all-cause mortality were 0.660 (*P* < 0.001), 0.647 (*P* < 0.001), and 0.604 (*P* = 0.017), respectively. The AUCs of NPAR, neutrophil percentage and albumin for 1-year all-cause mortality were 0.635 (*P* < 0.001), 0.619 (*P* < 0.001), and 0.595 (*P* = 0.0012), respectively. Similarly, the AUCs of NPAR, neutrophil percentage and albumin for 2-year all-cause mortality were 0.626 (*P* < 0.001), 0.599 (*P* < 0.001), and 0.601 (*P* = 0.017), respectively. The comparisons of ROC curves found NPAR was a better predictor than either albumin or neutrophil percentage, alone.

## Discussion

Our main findings are summarized as follows. First, a higher admission NPAR was association with worse clinical outcomes, including 90-day all-cause mortality, 1-year all-cause mortality and 2-year all-cause mortality in patients with CHF. Second, admission NPAR was proved as an independent predictor of short and long clinical outcomes in CHF patients, especially for HFpEF patients, after adjustments for several confounders. Third, ROC curves revealed that the admission NPAR had a better ability to predict all-cause mortality in patients with CHF, than either albumin or neutrophil percentage alone. To our knowledge, our study is the first to explore both short and long-term prognostic value of NPAR in patients with CHF.

The occurrence and development of HF is a complex pathophysiological process. Inflammation is a fundamental and persistent mechanisms involved in HF. Neutrophils are the most abundant type of white blood cells in peripheral blood of most mammals. And neutrophils are important factors of the innate immune system, which can coordinate inflammation-resolution and host defense mechanisms [19].

In the early stages of cardiac damage or infection, neutrophils, as key effector cells, are the first responders to clear deceased, ischemic myocyte debris or invasive pathogenic organisms from myocarditis. However, if short-lived neutrophils stay or migrate at the infarction site longer than normal, after heart injury or infection, dead neutrophils can release granular components into the extracellular environment, prolongating the ongoing inflammatory response and promoting advanced HF [20]. Besides, Tang [21] et all found that chronic angiotensin II infusion activated the neutrophil KLF2/NETosis pathway, triggering sporadic thrombosis in small myocardial vessels, leading to myocardial hypoxia and hypertrophy. The immunothrombotic dysregulation may be another mechanism for neutrophil-induced HF.

In the clinical setting, it is often observed that patients with AMI and AHF have significantly elevated levels of neutrophils in their peripheral blood at an early stage. In addition, higher neutrophil levels within the first 12 h after AMI predicted the occurrence of CHF and were associated with poor outcomes in AMI patients [22, 23]. Yang [24] et al. found peripheral blood neutrophil-to-lymphocyte ratio (NLR) was an independent predictive factor for MACE in the elderly patients with CHF and AF, which was similar to this study.

Albumin has long been regarded as an indicator of the body’s nutritional status. Also confirmatory evidence is that serum albumin exerts anti-inflammatory [6], antioxidant [25], anticoagulant and antiplatelet aggregation activity [7], which can join in several cardiovascular diseases. Prevalence of hypoalbuminemia ranges from 20 to 25% in CHF. And studies have shown that hypoalbuminemia is not only an independent predictor but also a powerful prognostic factor in CHF, mainly as a result of malnutrition and inflammation. A study including 5,795 older adults who were followed for 9.6 years, found that the occurrence of new onset HF was independently associated with hypoalbuminemia [26]. Occurrence of new onset HF was significantly related to low serum albumin concentration after adjusting for age, ejection fraction, renal function, inflammation, BP, diabetes and clinical presentation in 7192 patients with acute coronary syndrome [27].

Currently, lots of evidences confirm that hypoalbuminemia predicts adverse out-come independent of BMI, inflammation and liver function. Su [28] et al. found the low serum albumin level was association with adverse outcome in patients with systolic heart failure after adjusting for some traditional risk factors. Bonilla-Palomas [29] et al. found that hypoalbuminemia was a strong predictor for in-hospital and long-term mortality, after adjusting C-reactive protein, BMI, nutritional status and liver function, in both acute systolic and diastolic heart failure patients followed for 20 months.

As a combination of above two classical clinical evaluation parameters, NPAR was proved to be an independent predictor for clinical outcomes of many diseases, such as STEMI, severe sepsis and acute kidney injury [12, 14, 15]. Moreover, Yu et al. [13] found admission NPAR was independently associated with in-hospital, 30-day and 365-day mortality in patients with CS. And Hu et al. [30] found that NPAR was independently associated with in-hospital, 30-day, 90-day and 365-day mortality in patients with HF, including both acute heart failure (AHF) and CHF. All the above results were similar to our study. To our knowledge, this is the first study to explore the effect of NPAR for the short-term and long-term prognosis (2-year mortality) in patients with CHF. Further exploring the association between NPAR and three classifications of CHF patients, we found that elevated NPAR was associated with poor outcomes in patients with HFpEF, but not HFrEF. It is a result that was first discovered and it was actually sound from pathophysiological standpoint. Old concept of HFpEF was a hypertrophied heart with diastolic failure that evolves into systolic failure over time. However, prevailing concepts of HFpEF and HFrEF were separate diseases. Pathophysiological mechanism of HFpEF was mainly caused by microvascular inflammation and HFrEF was caused by cardiomyocyte loss associated with neurohormonal dysactivation [31]. Also, HFpEF was characterized in many patients by the coexistence of a systemic metabolic or inflammatory disorder that causes coronary endothelial dysfunction, microvascular rarefaction, and cardiac fibrosis [32]. The pathophysiological mechanism of HFpEF patients was more related to inflammation. So NPAR, as a useful marker of inflammation, can predict the clinical prognosis of HFpEF patients better than that of HFrEF patients.

In our study, we found that although both neutrophil percentage and albumin could influence the outcomes of patients with CHF, NPAR may offer more predictive power than the single factor. From ROC curves, the AUC used by NPAR to predict mortality in patients with severe CHF was greater than those used by neutrophil percentage and albumin, separately. In clinic, both neutrophil percentage and albumin are readily available, inexpensive and convenient. NPAR, combined with these two factors, may provide a fast assessment of risk for each patient with CHF in order to make a more precise decision for therapeutic strategy and medical resource allocation. Of course, according to the ROC analysis in our study, the predictive performance of NPAR for all-cause mortality in patients with CHF appears not good. Our findings suggest that a single NPAR test alone may also not be sufficient to predict long-term mortality in patients with CHF. However, whether NPAR can be combined with other symptom scores or traditional biomarkers to enhance prediction remains to be investigated.

### Limitation

Some limitations should be mentioned. This study was a single retrospective study; inevitable bias may affect the authenticity of the results. Second, although we have done our best to control for bias using multivariate models, there are still missing influence factors or other unknown factors that may confound the results. Furthermore, In addition, considering the unsatisfactory ROC values of NPAR, it is necessary to develop multivariate models or scoring systems that incorporate NPARs, in order to better predict clinical outcomes in patients with CHF.

## Conclusions

Admission NPAR is independently correlated with 90-day, 1-year and 2-year all-cause mortality in patients with CHF. Early evaluation of NPAR may help with risk stratification in patients with CHF. However, the predictive performance of NPAR alone on long-term mortality appears not good. In future, a prospective study are highly recommended to verify the prognostic value of NPAR combined with other predictors.

## Data Availability

The datasets used and/or analyzed during the current study are available from the corresponding author on reasonable request.

## Abbreviations

NPAR: neutrophil percentage-to-albumin ratio
HF: heart failure
CHF: chronic heart failure
STEMI: ST-segment elevation myocardial infarction
CS: cardiogenic shock
AKI: acute kidney injury
NYHA: New York Heart Association
NT-proBNP: N-terminal pro brain natriuretic peptide
LVEF: left ventricular ejection fraction
ESC: European Society of Cardiology
HFpEF: heart failure with preserved ejection fraction
HFmrEF: heart failure with mid-range ejection fraction
HFrEF: heart failure with reduced ejection fraction
CAD: coronary artery disease
AF: atrial fibrillation
SBP: systemic blood pressure
DSP: diastolic blood pressure
HR: heart rate
ACEI: angiotensin-converting enzyme inhibitor
ARB: angiotensin receptor blocker
cTnI: cardiac troponin I
Glu: glucose
Cr: creatinine
TG: triglycerides
TC: total cholesterol
LDL-c: low-density lipoprotein-cholesterol
hs-CRP: hypersensitive C-reaction protein
KM: Kaplan-Meier
HR: hazard ratios
CI: confidence intervals
CKD: chronic kidney disease
PCI: percutaneous transluminal coronary intervention
CABG: coronary artery bypass grafting
ROC: Receiver operating characteristics
AUC: area under the curve
UA: uric acid
BMI: body mass index
HDL-C: high-density lipoprotein cholesterol
LVEDD: left ventricular end-diastolic dimension
LVESD: left ventricular end-systolic dimension
MRA: mineralocorticoid receptor antagonist
NLR: neutrophil-to-lymphocyte ratio
AHF: acute heart failure
